# Supplementary material for: Gynecologic health of women with multiple sclerosis: An overview on the current status and findings of Pap tests in a low-income setting
Source: PLoS One. 2025 Mar 28;20(3):e0320069. doi: 10.1371/journal.pone.0320069 (PMC11952252; doi:10.1371/journal.pone.0320069)
Supplement: Attachment — Submitted filename: my comments.pdf [file pone.0320069.s001.pdf]

Thanks for inviting me to review this manuscript of PLOS jornal:

1. Some claims made in the discussion and introduction sections are unsupported by references. Please provide adequate citations to strengthen the credibility of these statements. For instance:

   ***Introduction**:*

- For instance, HPV vaccination could be offered to the wwMS if they are shown to bear an additional risk for HPV infection. Other instances could be offering behavioral consultation, sanitary products, as well as routine screening for, and treating of infections in both wwMS and their partners, if they are shown to bear an additional risk for such diseases.

   Discussion:

- Interestingly, a recent Mendelian randomization study on the UK biobank cohort found an association between MS and related factors (e.g., use of DMT) with cervical cancer but none of the other 14 investigated cancers.
- These results may be explained by an impairment in the immune response to cervical pathogens, possibly mediated by DMT.

- Furthermore, higher-grade inflammatory changes were more frequent in wwMS treated with aCD20 – a DMT class acting mainly on B cells.
- Another explanation could be the impairment of afferent pathways in wwMS. Particularly, impairment of afferent pathways in the wwMS with infection could render them asymptomatic.

2. About income:

- What is the methodology or scale for classifying income levels into mild, moderate, or severe categories?

- One critical aspect that required evaluation was the correlation between a patient's income level and their genital health. Unfortunately, this analysis is absent from the results and discussion sections. The study's methodology primarily just focused on matching MS patients with a control group for comparative analysis. → (Pap test results of wwMS were compared with a 1:2 age- and socioeconomic status (SES)-matched group of healthy controls (HC) who underwent Pap tests for cervical cancer screening in the same laboratory and in the same period)

3. Please specify in the methods section who provided the Pap smear samples: a gynecologist or another healthcare professional. This information is crucial for ensuring the adequacy and quality of the samples.

4. In certain instances please provide both the numerical count and the corresponding percentage together.

5. To enhance relevance, consider relocating this sentence ((Further of note, among all, only one of the wwMS with evidence of yeast infection showed severe inflammatory changes, whereas all HC with evidence of infection showed severe inflammatory changes)) and adding to the subsequent sentence ((As seen, the observed reactive/inflammatory changes differed between the participants with MS and matched HC; such changes were less frequently observed in the wwMS (OR: 0.3; 95% CI: 0.2, 0.4; $p < 0.001$)).

6. When analyzing (Pap test results), it would be insightful to rank DMTs based on their associated odds ratios (ORs), from highest to lowest. This ranking would facilitate the identification of DMTs potentially linked to increased risk of infection or inflammatory changes in MS patients. Also for better understanding you can mention as sentences which of DMT can cause more infection or inflammatory reaction?

7. In paragraph 3 of the discussion, please add the word (neural) before (afferent pathway) for better understanding.

8. please add (Benign) word before all phrases (inflammatory/reactive changes) for more comprehension.

9. As comorbidities such as diabetes can significantly impact susceptibility to infection in MS patients, it is crucial to explore the relationship between specific comorbidities and the risk of infection in wwMS. please explain the relationship or impact of comorbidities on infection in wwMS.

10. please mention the mean (SD) or median of **duration of DMT**.

11. Please provide more detailed information on the prevalence of Human Papillomavirus (HPV) infection in wwMS. If the prevalence of HPV is elevated compared to the general population, please elucidate the potential factors contributing to this increased risk. Additionally, please discuss the cervical malignancy presentation associated with HPV infection in MS patients.
