# Supplementary material for: Gynecologic health of women with multiple sclerosis: An overview on the current status and findings of Pap tests in a low-income setting
Source: PLoS One. 2025 Mar 28;20(3):e0320069. doi: 10.1371/journal.pone.0320069 (PMC11952252; doi:10.1371/journal.pone.0320069)
Supplement: Attachment — Submitted filename: R2R.docx [file pone.0320069.s002.docx]

Dear Editor,

Thank you for your consideration of our manuscript. We have considered all the editorial and reviewer comments, and revised our manuscript accordingly. Below, a point-by-point response to the editorial and reviewer comments could be found highlighted in green. We are confident that the revisions have increased the publication merit of the manuscript.

Once again, thank you for your consideration. We hope to hear from you soon.

Best regards

The authors

## Journal Requirements

1. When submitting your revision, we need you to address these additional requirements.

Please ensure that your manuscript meets PLOS ONE's style requirements, including those for file naming. The PLOS ONE style templates can be found at

https://journals.plos.org/plosone/s/file?id=wjVg/PLOSOne_formatting_sample_main_body.pdf and

https://journals.plos.org/plosone/s/file?id=ba62/PLOSOne_formatting_sample_title_authors_affiliations.pdf

**Response:** We ensured the revised files meet the style requirements as described in the mentioned templates.

2. We note that you have indicated that there are restrictions to data sharing for this study. For studies involving human research participant data or other sensitive data, we encourage authors to share de-identified or anonymized data. However, when data cannot be publicly shared for ethical reasons, we allow authors to make their data sets available upon request. For information on unacceptable data access restrictions, please see http://journals.plos.org/plosone/s/data-availability#loc-unacceptable-data-access-restrictions.

Before we proceed with your manuscript, please address the following prompts:

a) If there are ethical or legal restrictions on sharing a de-identified data set, please explain them in detail (e.g., data contain potentially identifying or sensitive patient information, data are owned by a third-party organization, etc.) and who has imposed them (e.g., a Research Ethics Committee or Institutional Review Board, etc.). Please also provide contact information for a data access committee, ethics committee, or other institutional body to which data requests may be sent.

b) If there are no restrictions, please upload the minimal anonymized data set necessary to replicate your study findings to a stable, public repository and provide us with the relevant URLs, DOIs, or accession numbers. Please see http://www.bmj.com/content/340/bmj.c181.long for guidelines on how to de-identify and prepare clinical data for publication. For a list of recommended repositories, please see https://journals.plos.org/plosone/s/recommended-repositories. You also have the option of uploading the data as Supporting Information files, but we would recommend depositing data directly to a data repository if possible.

Please update your Data Availability statement in the submission form accordingly.

**Response:** The contact information for the respective ethics committee was added in the revised manuscript. The Data Availability statemen was updated in the manuscript and submission form accordingly.

3. We note that your Data Availability Statement is currently as follows: All relevant data are within the manuscript and its Supporting Information files in aggregate form.

Please confirm at this time whether or not your submission contains all raw data required to replicate the results of your study. Authors must share the “minimal data set” for their submission. PLOS defines the minimal data set to consist of the data required to replicate all study findings reported in the article, as well as related metadata and methods (https://journals.plos.org/plosone/s/data-availability#loc-minimal-data-set-definition).

For example, authors should submit the following data:

- The values behind the means, standard deviations and other measures reported;

- The values used to build graphs;

- The points extracted from images for analysis.

Authors do not need to submit their entire data set if only a portion of the data was used in the reported study.

If your submission does not contain these data, please either upload them as Supporting Information files or deposit them to a stable, public repository and provide us with the relevant URLs, DOIs, or accession numbers. For a list of recommended repositories, please see https://journals.plos.org/plosone/s/recommended-repositories.

If there are ethical or legal restrictions on sharing a de-identified data set, please explain them in detail (e.g., data contain potentially sensitive information, data are owned by a third-party organization, etc.) and who has imposed them (e.g., an ethics committee). Please also provide contact information for a data access committee, ethics committee, or other institutional body to which data requests may be sent. If data are owned by a third party, please indicate how others may request data access.

**Response:** As mentioned, the manuscript contains all relevant “aggregate” data, and does not include “raw” data. The raw data contain potentially identifying information pertaining to the participants of the study. Thus, the contact information for the respective ethics committee was added in the revision. The Data Availability statemen was updated in the submission form accordingly.

## Additional Editor Comments:

“Thank you for submitting your manuscript entitled "Gynecologic health of women with multiple sclerosis: An overview on the current status and findings of Pap tests in a low-income setting." You have selected a highly relevant and underexplored topic that holds significant importance in the literature.

However, after a thorough review, I have identified several essential aspects that need to be addressed to enhance the clarity, depth, and impact of your manuscript. Please refer to the detailed comments and suggestions provided to guide your revisions. Addressing these points will significantly strengthen the manuscript's contribution to the field.”

**Response:** Thank you for dedicating your time to our manuscript. We are confident that the revision has added to the publication merit of our manuscript.

# Reviewer's Responses to Questions

1. Is the manuscript technically sound, and do the data support the conclusions?

The manuscript must describe a technically sound piece of scientific research with data that supports the conclusions. Experiments must have been conducted rigorously, with appropriate controls, replication, and sample sizes. The conclusions must be drawn appropriately based on the data presented.

Reviewer #1: Yes

Reviewer #2: Yes

**Response:** We would like to thank both of the reviewers for acknowledging the technical soundness of our study and the appropriateness of the conclusions.

2. Has the statistical analysis been performed appropriately and rigorously?

Reviewer #1: Yes

Reviewer #2: Yes

**Response:** We would like to thank both of the reviewers for acknowledging the appropriateness and rigor of the statistical methods.

3. Have the authors made all data underlying the findings in their manuscript fully available?

The PLOS Data policy requires authors to make all data underlying the findings described in their manuscript fully available without restriction, with rare exception (please refer to the Data Availability Statement in the manuscript PDF file). The data should be provided as part of the manuscript or its supporting information, or deposited to a public repository. For example, in addition to summary statistics, the data points behind means, medians and variance measures should be available. If there are restrictions on publicly sharing data—e.g. participant privacy or use of data from a third party—those must be specified.

Reviewer #1: Yes

Reviewer #2: No

**Response:** The data produced during the presented study is presented in aggregate form. Raw data is not included in the submission. The raw data contains potentially identifying information pertaining to the participants of the study, thus, its public, unrestricted sharing may raise ethical issues. We added the contact details of the research ethics committee to the revised manuscript.

4. Is the manuscript presented in an intelligible fashion and written in standard English?

PLOS ONE does not copyedit accepted manuscripts, so the language in submitted articles must be clear, correct, and unambiguous. Any typographical or grammatical errors should be corrected at revision, so please note any specific errors here.

Reviewer #1: Yes

Reviewer #2: Yes

**Response:** We would like to thank both of the reviewers for acknowledging the clarity of the manuscript’s language.

# Review Comments to the Author

## **Reviewer #1**

*“This manuscript titled; Gynecologic health of women with multiple sclerosis: An overview on the current status and findings of Pap tests in a low-income setting*

*Thank you for your valuable contribution to the field. Early diagnosis of gynecological issues is critical for patients with MS, and the authors have provided an analysis to highlight this in their tables.*

*To further enhance the value of this manuscript, I would like to suggest additional points to help strengthen it.”*

**Response:** Thank you for dedicating your time to our manuscript. We endeavoured to address your comments in a clear and comprehensive fashion, and believe doing so has improved the quality of our manuscript significantly.

*“Below are my comments for improvement:*

*Methods:*

*The authors haven't reported the total number of pregnancies in patients with MS, which may have a significant impact on the course of MS, particularly concerning the EDSS and disease progression.”*

**Response:** Thank you for this comment. Indeed, each participant’s pregnancies could have affected MS outcomes. As our outcome was not disability worsening, we regretfully did not collect information regarding the number of prior pregnancies of participants. We explicitly acknowledged this point as a limitation so that future studies could bear this particular point in mind (limitations paragraph in discussion section).

*“However, the manuscript does not specify the MS subtypes in the patients, such as relapsing-remitting (RR), primary progressive (PP), or secondary progressive (SP) MS. This lack of stratification is important, as the type of MS could influence both disease progression and potentially the results of Pap smears.”*

**Response:** Thank you for your comment. The third line of the first paragraph of the “results: characteristics of participants” section reads:

*“At the time of the study, 18 (9.1%) had secondary progressive, while the others had relapsing-remitting MS.”*

As interpreted, no person with primary progressive MS participated in the study. This was mentioned as a limitation in the revised manuscript (limitations paragraph in discussion section).

*“It would be valuable for the authors to include an analysis of the MS subtype with Pap smear results, as this could reveal important patterns or associations. I would also suggest including a detailed analysis of the relationship between MS subtype and the Pap smear results, as this would offer more insight into the possible interactions between disease type and cervical health.”*

**Response:** Thank you for allowing us to clarify. The last paragraph of the “3.3. Pap test results” subsection of the “Results” section reads:

*“Furthermore, using ordinal and binary logistic regression, the association of the pap test results (i.e., the outcome variables mentioned above) was investigated with the covariates age, MS duration and subtype, total number of MS relapses, total number of times undergoing corticosteroid pulse therapy, EDSS score, historical and current DMT and duration thereof. A positive association was found between current aCD20 therapy and the degree of benign inflammatory/reactive changes (unadjusted OR vs. interferons: 2.7; 95% CI: 1.1, 6.8; p = 0.03). This association was seen multivariable analysis as well (adjusted OR vs. interferons: 3.5; 95% CI: 1.3, 9.1; p = 0.01). Other results returned unremarkable.”*

As mentioned, MS subtype was already included as a covariate in the regression analyses, returning unremarkable results. This could be due to the fact that the study lacked people with primary progressive MS and had a low count of participants with secondary progressive MS. We acknowledged this in the revised discussion section (limitations paragraph in discussion section).

*“Additionally, in the exclusion criteria, patients with MS who have had intercourse within 48 hours of the Pap smear should be excluded from the study. This recommendation is based on established guidelines in gynecological research, as recent intercourse may interfere with test accuracy by causing irritation or altering the cells on the cervix. The authors should address this consideration more explicitly in the study design.”*

**Response:** Thank you for mentioning. The sixth inclusion criterion of the study reads:

“absence of any medical, cultural, and/or ethical contraindication for Pap testing.”

We acknowledge the ambiguity of this sentence; thus, the revised sentence now features examples of considered “contraindications” to enable most accurate comprehension by the readers.

*“Tables:*

*Each table should have a clear and concise title placed above the table. Additionally, all abbreviations used within the tables should be listed below the table for clarity and ease of reference.”*

**Response:** Thank you for your suggestion. The revised tables now feature a clear and concise title above, and a list of all abbreviations (in order of being mentioned in the table) below.

*“The percentages in each column should add up to exactly 100%, with no exceptions, to ensure accuracy in data presentation.”*

**Response:** Thank you for mentioning. We noticed that due to rounding of the percentages in some instances, the percentages in some columns adds up to 100% +/- 0.2% instead of exactly 100%. We revised the rounding so that all percentages add up to 100% in all tables. Additionally, the following revisions were made in Table 1: (i) the count and percentage for those not reporting any method for contraception was added, and erroneous percentages in this section were corrected; (ii) the count and percentage of ones reporting no comorbidity was added. Please note, some persons reported having multiple comorbidities, so the percentages in the comorbidities section correctly exceeds 100%. (iii) erroneous count and percentages in the “usage of vaginal cleansing products” section was corrected. In Table 2: (i) erroneous count of healthy controls without evidence of infection was corrected.

*“Furthermore, demographic data for the HC group are currently missing, I mean that all of the demographic data for MS patients in the first table must be provided for the HC group, too. This information is essential for making meaningful comparisons between the MS patients and the HC group. Including demographic data for both groups in the tables and within the body of the manuscript would enhance the comprehensibility and rigor of the study's findings.”*

**Response:** Thank you for mentioning your concern. As mentioned in the manuscript, the healthy control group consisted of people who referred to the same laboratory in the same period; the data of whom was collected retrospectively from the laboratory records. The laboratory records featured basic data to confirm eligibility of the healthy controls (e.g., age, socioeconomic status, symptoms, reason for referral, etc.), yet, at the time of the study, we could not gain access to their contact information. Thus, the healthy controls were not surveyed, and the survey data (i.e., information presented in Table 1) is not available for the healthy controls. This was explicitly stated as a limitation (limitations paragraph in discussion section); we are hoping future investigators account for this point through reading our manuscript.

## **Reviewer #2**

*“1. Some claims made in the discussion and introduction sections are unsupported by references. Please provide adequate citations to strengthen the credibility of these statements.”*

**Response:** Thank you for noticing, and apologies for overlooking such errors. We made sure all the references are added to the manuscript.

*“For instance:*

*Introduction:*

**For instance, HPV vaccination could be offered to the wwMS if they are shown to bear an additional risk for HPV infection. Other instances could be offering behavioral consultation, sanitary products, as well as routine screening for, and treating of infections in both wwMS and their partners, if they are shown to bear an additional risk for such diseases.”*

**Response:** References were added.

*“Discussion:*

**Interestingly, a recent Mendelian randomization study on the UK biobank cohort found an association between MS and related factors (e.g., use of DMT) with cervical cancer but none of the other 14 investigated cancers.”*

**Response:** Reference was added.

*“*These results may be explained by an impairment in the immune response to cervical pathogens, possibly mediated by DMT.”*

**Response:** This statement is made based on the general immunomodulatory effects of DMT; we could not find any study directly linking DMT to impairments in cervical mucosal immune responses to pathogens. We clarified in the revised manuscript.

*“*Furthermore, higher-grade inflammatory changes were more frequent in wwMS treated with aCD20 – a DMT class acting mainly on B cells.”*

**Response:** This has been an observation made in the present study. We clarified in the revised manuscript.

*“*Another explanation could be the impairment of afferent pathways in wwMS. Particularly, impairment of afferent pathways in the wwMS with infection could render them asymptomatic.”*

**Response:** References were added.

*“2.About income:*

**What is the methodology or scale for classifying income levels into mild, moderate, or severe categories?”*

**Response:** Thank you for asking, enabling us to clarify. Income was self-reported by participants. Classification was based on the income figures reported by the national statistics center of Iran – a government organization which carries out nationwide sociodemographic studies on the Iranian population. This clarification was added (methods: variables and measurements section).

*“*One critical aspect that required evaluation was the correlation between a patient's income level and their genital health. Unfortunately, this analysis is absent from the results and discussion sections. The study's methodology primarily just focused on matching MS patients with a control group for comparative analysis. → (Pap test results of wwMS were compared with a 1:2 age- and socioeconomic status (SES)-matched group of healthy controls (HC) who underwent Pap tests for cervical cancer screening in the same laboratory and in the same period)”*

**Response:** Indeed. Thank you for pointing out. Numerous studies from prior decades exist pointing towards an association between SES and Pap test results (e.g., PMID: 22281605, PMID: 17119040, etc.) We as well, did this analysis per your suggestion. However, results returned unremarkable. This point was added to the revised manuscript (last paragraph of the results section).

*“3. Please specify in the methods section who provided the Pap smear samples: a gynecologist or another healthcare professional. This information is crucial for ensuring the adequacy and quality of the samples.”*

**Response:** Requested detail added to the revised manuscript in the methods: population and setting section.

*“4. In certain instances please provide both the numerical count and the corresponding percentage together.”*

**Response:** We made sure the numerical counts are presented along corresponding percentages in all instances in the revised manuscript.

*“5. To enhance relevance, consider relocating this sentence ((Further of note, among all, only one of the wwMS with evidence of yeast infection showed severe inflammatory changes, whereas all HC with evidence of infection showed severe inflammatory changes)) and adding to the subsequent sentence ((As seen, the observed reactive/inflammatory changes differed between the participants with MS and matched HC; such changes were less frequently observed in the wwMS (OR: 0.3; 95% CI: 0.2, 0.4; p < 0.001)).”*

**Response:** The mentioned sentence was relocated per your suggestion (results: Pap test results section).

*“6. When analyzing (Pap test results), it would be insightful to rank DMTs based on their associated odds ratios (ORs), from highest to lowest. This ranking would facilitate the identification of DMTs potentially linked to increased risk of infection or inflammatory. changes in MS patients. Also for better understanding you can mention as sentences which of DMT can cause more infection or inflammatory reaction?”*

**Response:** Thank you for giving us this idea. We decided to add a figure where the effect measure of each DMT is visualized, facilitating the comparison of the DMTs in terms of their effect on the outcomes (new figure 1).

*“7. In paragraph 3 of the discussion, please add the word (neural) before (afferent pathway) for better understanding.”*

**Response:** The word “neural” was added to the phrase “afferent pathway” in the revised manuscript (lines 275-279 in the discussion section).

*“8. please add (Benign) word before all phrases (inflammatory/reactive changes) for more comprehension.”*

**Response:** The word “benign” was added before all of the “inflammatory/reactive changes” phrases in the revised manuscript.

*“9. As comorbidities such as diabetes can significantly impact susceptibility to infection in MS patients, it is crucial to explore the relationship between specific comorbidities and the risk of infection in wwMS. please explain the relationship or impact of comorbidities on infection in wwMS.”*

**Response:** Indeed. Thank you for mentioning. Comorbidities were added to our regression analyses. It should be noted, however, data on comorbidities were based on reports of participants themselves, raising validity issues (e.g., diagnoses such as DM and/or HTN were not objectively confirmed by the investigators). Additionally, the three wwMS with non-benign cellular changes did not report having any comorbidity, thus, the association of comorbidities with non-benign cellular changes could not be investigated. These points were explicitly mentioned in the revised manuscript (last paragraph of results section, and the limitations paragraph in the discussion section.)

*“10. please mention the mean (SD) or median of duration of DMT.”*

**Response:** The information pertaining to the duration of exposure to each DMT was added to the revised manuscript (results: Characteristics of participants section.)

*“11. Please provide more detailed information on the prevalence of Human Papillomavirus (HPV) infection in wwMS. If the prevalence of HPV is elevated compared to the general population, please elucidate the potential factors contributing to this increased risk. Additionally, please discuss the cervical malignancy presentation associated with HPV infection in MS patients.”*

**Response:** As far as we know, there is currently insufficient evidence available in terms of the comparative risk of HPV infection in wwMS and the general population. A consensus statement in 2021 (PMID: 32940121) reads: *“4.1 No data on the association between MS and the natural history of HPV infection and progression to pre-invasive and invasive diseases are available (grade of evidence: A1c.)”* A more recent review study (PMID: 36846149) also failed to find a documentation of this comparative risk in the literature. This point was mentioned in the revised manuscript (lines 59-61 in the introduction, and lines 247-249 in the discussion sections.)

Additionally, we could not find any evidence indicating a different presenting symptomatology of cervical cancer in wwMS compared to general population. Yet, a delay in diagnosis may be plausible as symptoms may be attributed to MS-related issues, which is the reason more extensive gynecologic check-ups are recommended in wwMS (PMID: 32940121). This point was added to the revised manuscript (lines 244-247 in the discussion section.)
